# Design and Implementation of Arch Function for Adaptive Multi-Finger Prosthetic Hand

**DOI:** 10.3390/s19163539

**Published:** 2019-08-13

**Authors:** Xu Yong, Xiaobei Jing, Xinyu Wu, Yinlai Jiang, Hiroshi Yokoi

**Affiliations:** 1 Guangdong Provincial Key Laboratory of Robotics and Intelligent System, Shenzhen Institutes of Advanced Technology, Chinese Academy of Sciences (CAS), Shenzhen 518055, China; 2 CAS Key Laboratory of Human-Machine Intelligence-Synergy Systems, Shenzhen Institutes of Advanced Technology, Chinese Academy of Sciences (CAS), Shenzhen 518055, China; 3 Department of Mechanical Engineering and Intelligent Systems, The University of Electro-Communications, 1-5-1 Chofugaoka, Chofu, Tokyo 1828585, Japan; 4 SIAT Branch, Shenzhen Institute of Artificial Intelligence and Robotics for Society, Shenzhen 518055, China; 5 Center for Neuroscience and Biomedical Engineering, The University of Electro-communications, Tokyo 1828585, Japan; 6 Beijing Innovation Center for Intelligent Robots and Systems, Beijing 100081, China

**Keywords:** prosthetic hand, arch function, adaptive grasping, multi-finger

## Abstract

Although arch motions of the palm substantially contribute to frequent hand grasping, they are usually neglected in the development of prosthetic hands which focuses on digit movements. We designed the arch function for its implementation on an adaptive multi-finger prosthetic hand. The digits from the developed hand can perform adaptive grasping, and two carpometacarpal joints enable the palm of the prosthetic hand to form an arch with the thumb. Moreover, the arch posture can be passively released, mimicking the human hand switching between sphere and medium wrap grasps according to the situation. Other requirements such as weight, cost, and size limitations for hand prostheses were also considered. As a result, we only used three actuators fully embedded in the palm through a novel tendon-driven transmission. Although the prosthetic hand is almost the same size of an adult hand, it weighs only 146 g and can perform 70% of the 10 most frequent grasps.

## 1. Introduction

The hand is essential for humans to interact with the world. Its dexterous anatomy makes the hand a complex integrated system with high degrees of freedom (DOFs) that enable a vast variety of uses [1,2,3,4,5,6,7,8]. However, existing prosthetic hands cannot completely reach the complexity of the human hand, even with the currently available technology, given several limitations such as weight, appearance, packaging, maintenance, and operability [9,10,11,12,13].

**Figure 1:**
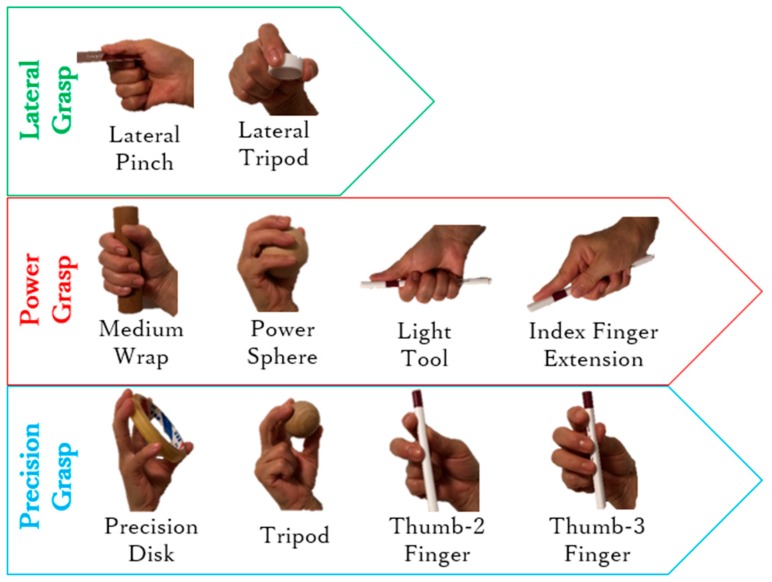
Ten most frequent grasps (adapted from Reference [13]).

**Figure 2:**
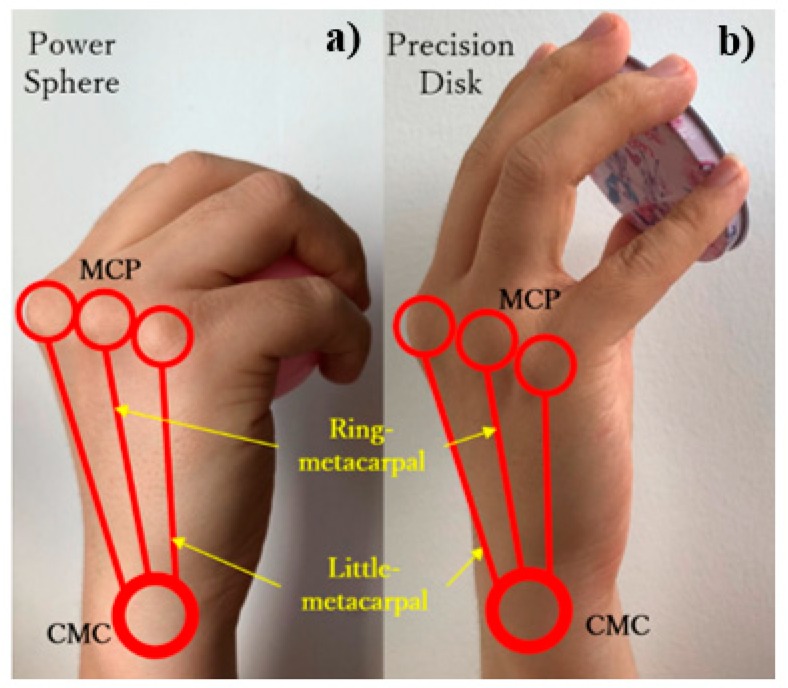
Adaptability during (**a**) power sphere and (**b**) precision disk grasps.

In this study, we focused on prehensile grasps with one hand to securely seize and partly or completely hold an object within the hand [14]. Still, prehensile grasps are vast and include several types. In fact, human grasping has been extensively investigated with several studies and surveys available. However, no consolidated categorization of grasping exists by the variety of research scopes and purposes [15,16,17,18]. The taxonomy developed by Feix et al. [15] is considered the most complete. Using this taxonomy, Bullock et al. identified the frequency of human grasps in some activities of daily living and extracted the ten most frequent grasp types, which account for approximately 80% of all the grasping tasks [13]. Overall, these grasp types can be divided into three main types: power, lateral, and precision grasps, as shown in Figure 1. Most existing prosthetic hands have been designed to perform these three main types of grasps, including various design concepts like fully actuated drives, underactuated drives, manually operated thumbs, and motor-actuated thumbs [19,20,21,22,23,24]. Nevertheless, these prosthetic hands usually focus on the DOFs from the digits that can complete most grasps but neglect the arch function of the hand due to considering the palm as a rigid body without metacarpal features [25,26,27,28,29,30]. The arch function allows the hand to wrap and to easily adapt around object shapes, especially when performing sphere grasps [1]. In the ten most frequent grasps (Figure 1), it is obvious that both the power sphere and precision disk grasps need sphere or circular enclosing. The human hand palm is formed by five metacarpal bones, each forming a carpometacarpal (CMC) joint together with the corresponding wrist bones. Through the conjunction with the flexion even rotation, the CMC joints allow the palm to form an arch, thus providing a sphere enclosing. Although the angular movements in the CMC joints are small, their slight change generates considerable digit translations. As the distance from the metacarpophalangeal (MCP) joint increases, digit translation is larger. For an average hand size, a 5° flexion of the CMC joint results in 10–15 mm translations of the fingertip [1], which allows the fingertips to easily from a circular enclosing. Hence, digits can notably contribute to adaptability during grasps through slight joint rotations, especially for power sphere and precision disk grasps, as shown in Figure 2. Therefore, the arch function of the palm considerably improves grasping, and including it in prostheses would resemble the human hand movement more naturally, thus enabling more varied grasp types. In addition, adding the arch function will retain much more biological features, which leads to be more effective and intuitive controlled prosthesis [31].

**Figure 3:**
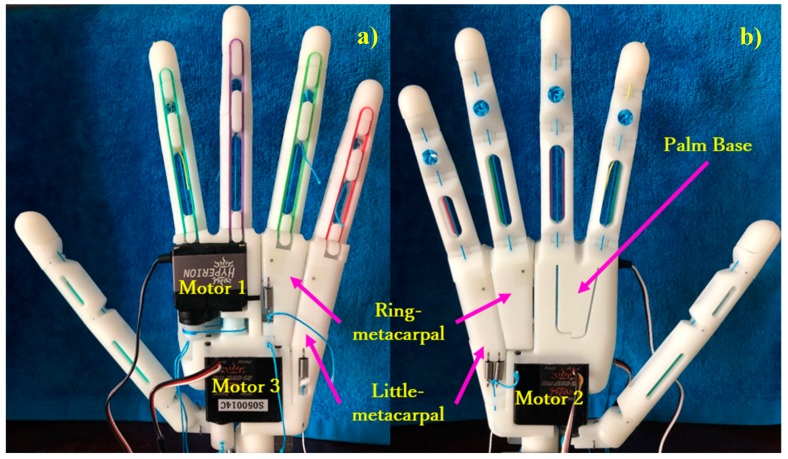
Assembled prosthetic hand: (**a**) back view and (**b**) front view.

In this study, we designed the arch function of the human hand and implemented it to an adaptive multi-finger prosthetic hand. The four fingers of the prosthesis provide a passive adaptive grasp, and the thumb can flex/extend or adduct/abduct independently. As shown in Figure 3, two metacarpals are included in the palm, which is not regarded as a rigid block. The metacarpals are connected to the ring (ring-metacarpal) and little (little-metacarpal) fingers independently and can passively flex/extend with a certain angle along with adduction of the thumb, thus establishing the palm arch. Furthermore, the metacarpals can be passively returned to their initial position, releasing the arch and closely resembling the human hand function during different grasps to adjust its posture. Although the proposed prosthetic hand has high DOFs from its 18 moveable joints, only three actuators are employed. As the main design constraints are weight, human-like appearance, and package size, the prosthetic hand should be suited for daily use by transradial amputees. Consequently, we integrated all the actuators and transmissions within the prothesis, which is approximately the size of an adult hand. Moreover, the weight of the prosthetic hand should remain below 370 g [32].

The design and mechanism of the five digits are outlined in Section 2. Section 3 details the arch function of the palm, including the kinematics and static modeling, as it is the main design consideration. Section 4 reports the experiments including hand motion verifications by using soft sensors and intuitive control implemented via electromyography (EMG) signals to perform an activity of daily living. Finally, we draw conclusions in Section 5.

## 2. Digit Design

**Figure 4:**
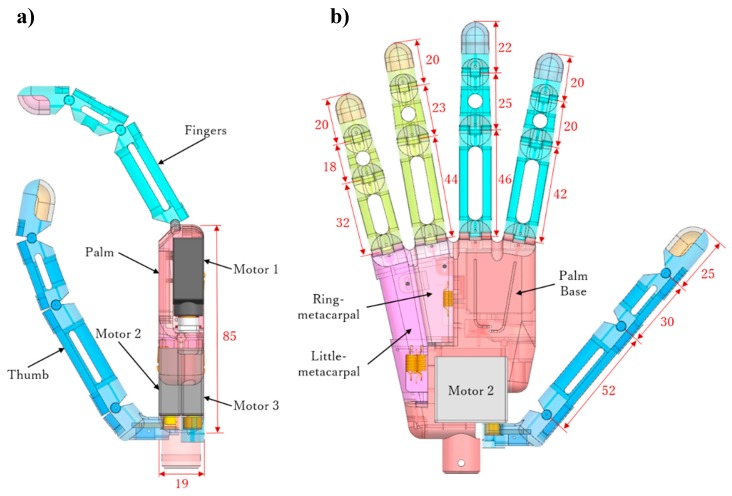
Three-dimensional (3-D) model of proposed prosthetic hand: (**a**) side view and (**b**) front view (unit, millimeters).

Figure 4 shows the 3-D model of the developed prosthetic hand, which consists of five digits connected to the palm. To naturally mimic hand grasps, we release the flexional joint of each digit like in the human hand. The four fingers share one actuator, motor 1 (HP-DS13-FMB, 5.5 kg/cm, Atlas Digital Servo, Hyperion, Hongkong, China), such that they work as a unit to perform flexions and extensions. The thumb is actuated by two motors, motors 2 and 3 (HP-DS095-FMD, 4.5 kg/cm, Atlas Digital Servo, Hyperion, Hongkong, China), endowing it with two independent DOFs for flexion/extension and adduction/abduction. In addition, given their compactness and compliance, tendon-driven transmissions are adopted for actuating all the digits.

### 2.1. Fingers

Every finger, except for the thumb, in the developed prosthetic hand consists of three segments, namely, proximal, intermediate, and distal phalanges, and three flexional joints, namely, MCP, proximal interphalangeal (PIP), and distal interphalangeal (DIP) joints. The phalange segments in a finger are connected through a shaft, as shown in Figure 4.

**Figure 5:**
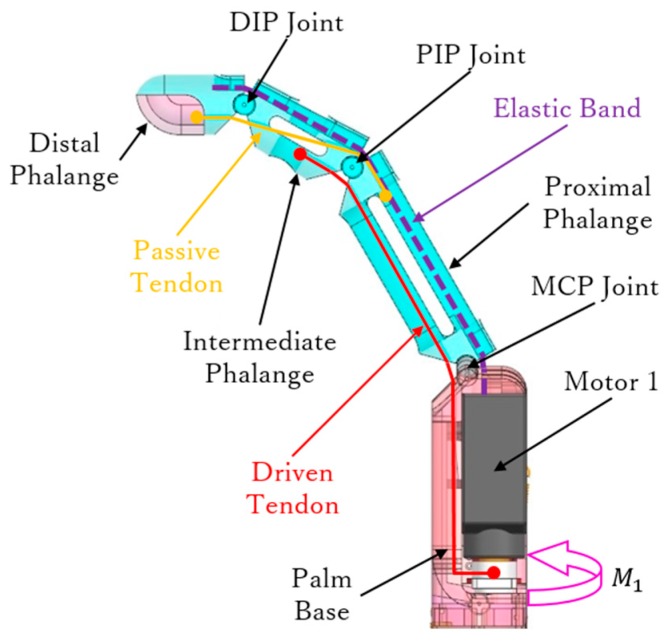
Side view of the 3-D model from an assembled finger.

As the four fingers have the same mechanical structure and driving method, we only provide the details of one finger, as shown in Figure 5. Two kinds of tendons are employed for different purposes, namely, driven and passive tendons. The driven tendon crosses through the MCP and PIP joints to then connect to motor 1. The passive tendon is fixed between the proximal and distal phalanges, passing through the PIP and DIP joints. These tendon configurations allow the finger to perform adaptive grasps and to determine grasping trajectory.

**Figure 6:**
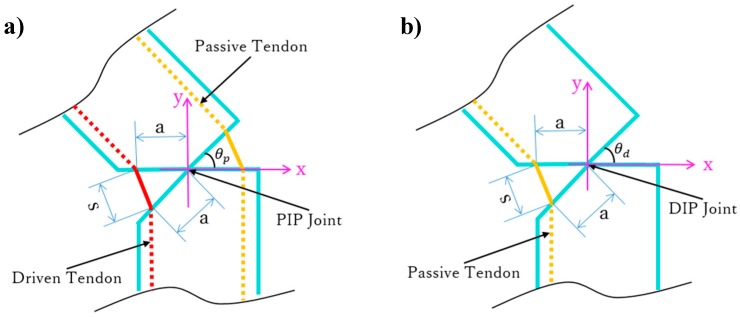
Geometry for tendon motion in the (**a**) proximal interphalangeal (PIP) and (**b**) distal interphalangeal (DIP) joints.

First, as motor 1 rotates (*M*_1_ in Figure 5), the driven tendon is rolled up to flex the MCP and PIP joints, thus providing adaptive flexion during grasps. In fact, the driven tendon induces flexion on the other phalanges until the object blocks motion on any or all the phalanges. Then, with the flexion of the PIP joint, the passive tendon is involved to flex the DIP joint together. The geometry of the PIP and DIP joints is shown in Figure 6. Taking the joint rotation axis as the origin, the driven and passive tendons are distributed symmetrically on both sides of the PIP joint. In Figure 6, $\theta_{p}$ is the angle of the PIP joint, a is the length constant between the origin and tendon, and *s* is the variable travel distance of the tendon. In the DIP joint, the same geometry is setup for the passive tendon. Thus, the passive tendon always travels the same distance *s* as the driven tendon, and hence, the rotation angle of the DIP joint, $\theta_{d}$, is equal to $\theta_{p}$. Therefore, the passive tendon ensures a determined trajectory of the DIP joint, which rotates along the joint axis with the same angular velocity as the PIP joint.

To perform the joint extension necessary for the fingers to return to their initial posture, an elastic band is embedded in the back of each finger, as shown in Figure 5. Hence, reverse rotation of motor 1 forces both the driven and passive tendons to be released and to become slack, and the finger joints extend according to the resilience force from the elastic band. However, an appropriate elastic band should be selected. In fact, a very small elastic constant fails to provide enough rebound force in the elastic band, whereas an excessive elastic constant reduces the motor output. We determined the appropriate elastic constant as follows.

**Figure 7:**
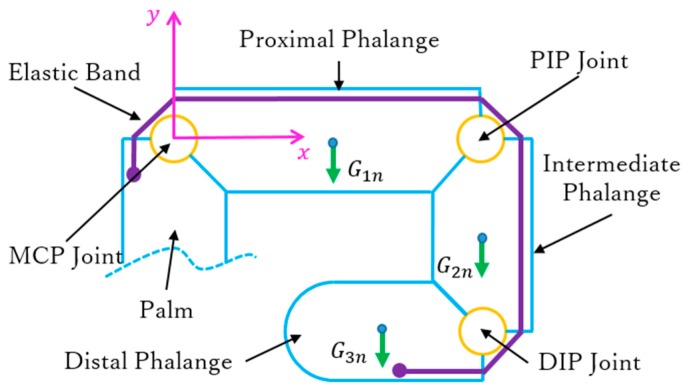
Statics diagram of fully flexed finger.

By considering the elastic band as a spring, the elastic constant for each finger can be represented as $k_{n}$ with n=1, 2, 3, 4 for the index, middle, ring, and little fingers, respectively. Figure 7 shows a fully flexed finger, a position from which the elastic band should overcome every phalange gravity force for driving the finger back to the fully extended position. This can be regarded as a boundary state because, if the elastic constant is small, the finger cannot return to the fully extended position, whereas if the elastic constant is large, excessive elastic resistance is generated during grasping.

Therefore, the elastic constant can be determined through the following equilibrium equation:

(1) $$\overset{3}{\underset{i=1}{\sum }}\int^{\;}-G_{in}dy=\overset{3}{\underset{j=1}{\sum }}\frac{1}{2}k_{n}{\left(x_{jn}-x_{0n}\right)}^{2}$$

where *i* = 1, 2, 3 corresponds to the proximal, intermediate, and distal phalanges, respectively, and where *j* = 1, 2, 3 corresponds to the MCP, PIP, and DIP joints, respectively. Hence, $G_{in}$ is the gravity force of the *i*th phalange on the *n*th finger, $x_{jn}$ is the maximum stretching on each joint of every finger, and $x_{0n}$ is the initial stretching corresponding to each $x_{jn}$.

### 2.2. Thumb

**Figure 8:**
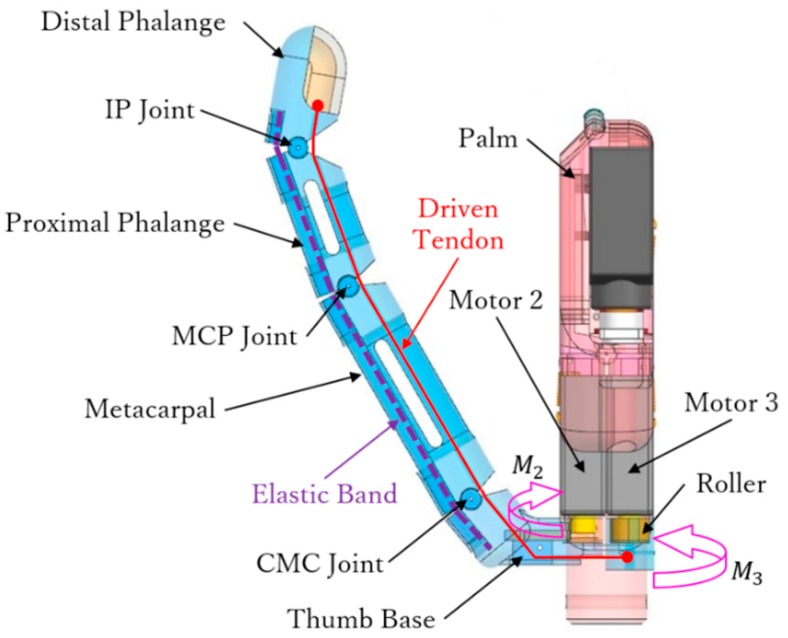
Tendon driven mechanism of the thumb.

The human thumb has a special function as it can move independently without involving other fingers. Figure 8 shows the thumb assembled to the palm base in the proposed prosthetic hand and the driven tendon mechanism. The thumb consists of four segments, namely, distal and proximal phalanges, metacarpal, and thumb base. Two motors provide the two DOFs of the thumb to perform flexion/extension and adduction/abduction.

Motor 2 generates adduction and abduction. As shown in Figure 8, the thumb is linked to motor 2 through the thumb base, and thus, this motor enables the thumb to rotate inward (adduction) and outward (abduction). The range of adduction/abduction in the thumb is 0–90°. Furthermore, motor 2 actuates the palm to form an arch, as detailed in Section 3.

**Figure 9:**
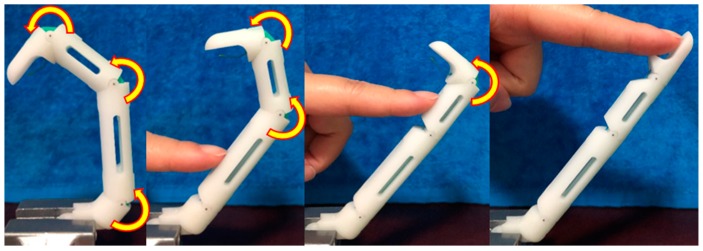
Adaptive grasping of the thumb.

Motor 3 generates flexion and extension of the thumb. As shown in Figure 8, the driven tendon crossing through the interphalangeal (IP), MCP, and CMC joints is fixed to motor 3 through a roller. Rotation of motor 3 makes the driven tendon move the thumb in flexion or release the joints. As the driven tendon crosses through all the joints, an adaptive grasp can be achieved, as illustrated in Figure 9.

Finally, an elastic band is arranged like for the four other fingers to extend the thumb, as shown in Figure 8. The elastic constant can be calculated as follows:

(2) $$\overset{3}{\underset{i=1}{\sum }}\int^{\;}-G_{i}dy=\overset{3}{\underset{j=1}{\sum }}\frac{1}{2}k{\left(x_{j}-x_{0}\right)}^{2}$$

where *i* = 1, 2, 3 corresponds to the metacarpal, proximal, and distal phalanges, respectively, and where *j* = 1, 2, 3 corresponds to the CMC, MCP, and IP joints. Hence, $G_{i}$ is the gravity force of the *i*th phalange, $x_{j}$ is the maximum stretching on each joint, and $x_{0}$ is the initial stretching corresponding to each $x_{j}$.

## 3. Arch Function

To realize the arch function of the hand, we include DOFs in the palm instead of regarding it as a rigid structure. Thus, the palm can form an arch with the thumb and allows the hand to adapt to object shapes during grasps, especially of the sphere type. Furthermore, the arch can be released passively, resembling the adjusting ability of the human hand to switch between sphere and medium wrap grasps.

### 3.1. Palm Design

**Figure 10:**
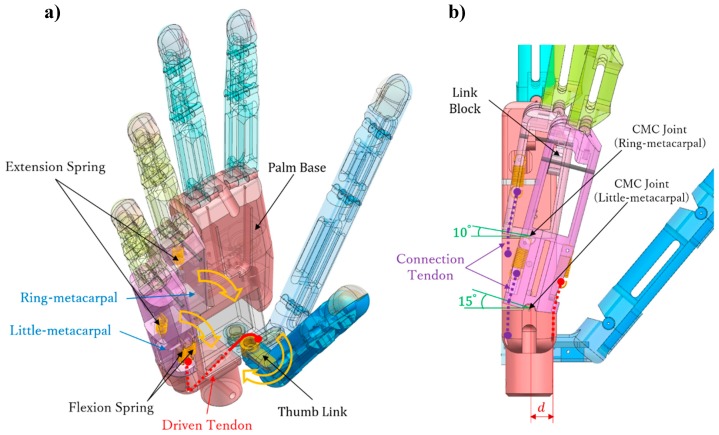
Three-dimensional model of the palm: (**a**) front view (final arch position) and (**b**) side view (final arch position).

We modeled the palm according to a human hand, with its size being 85 mm long, 70 mm wide, and 20 mm thick (Figure 4), and all the motors embedded on it. Figure 10 shows that the palm is divided into three parts, namely, palm base, ring-metacarpal, and little-metacarpal. The ring and little-metacarpals are connected to the palm base by shafts, which form the CMC joint with the palm base (Figure 10b). According to the human hand motion, we set the maximum flexion of the ring- and little-metacarpals to 10 and 15°, respectively [33].

A driven tendon and a symmetrical spring set are equipped in the metacarpals to realize the arch function. The symmetrical spring set consists of four springs symmetrically distributed on both sides of the CMC joints. Specifically, two springs are fixed on the front of the little-metacarpal (Figure 10a) and connected to motor 2 through the thumb link, establishing flexion springs, which are responsible for the flexion of the two metacarpals. The two other springs are fixed on the back of the two metacarpals, establishing extension springs, which are responsible for the extension of each metacarpal. The ring-metacarpal is coupled to the little-metacarpal through a link block (Figure 10b). Therefore, thumb adduction first produces little-metacarpal flexion, which after some degrees starts ring-metacarpal flexion. Finally, thumb adduction over 30° causes the two metacarpals to reach their maximum flexion to form an arch with the thumb. This process of arch forming sets a sphere grasp, in which the palm does not contact any object under no load. The metacarpals return to their initial posture (Figure 12b) and release the arch with thumb abduction. Figure 10a shows the front view with the metacarpals completely flexed to form an arch with the thumb, whereas Figure 10b shows the side view, representing the final arch position. At this position, d is the distance between the driven tendon to the CMC joint, and neither the little-metacarpal nor the ring-metacarpal flex to their maximum angle (15 and 10°) independently.

**Figure 11:**
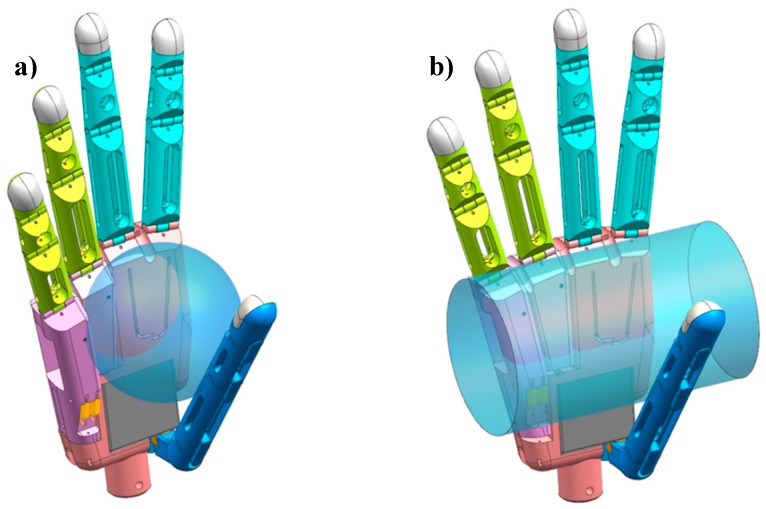
Holding objects using (**a**) spherical grasp and (**b**) medium wrap grasp.

After the metacarpals are flexed to the final arch position, the thumb can continue adducting because its maximum rotation is 90°. From this moment, assuming that motor 2 is no longer idle, an object is grasped inside the arch. With the rotation of the thumb, the metacarpals extend passively to the initial position due to the increasing action force from the contact surface. As a result, the arch can switch between the sphere and medium wrap grasps. Figure 11 illustrates the metacarpal extension to the initial position from spherical grasp to medium wrap grasp.

Similar to the selection of elastic band for the digits, the spring stiffness of the extension spring can be determined first. The sum over flexion spring stiffness should be equal to that over extension spring stiffness. In fact, if the flexion side outweighs the extension side, it obstructs thumb adduction, whereas the arch function cannot be realized otherwise. In other words, flexion and extension should be balanced when the palm is at the initial position. Therefore, the spring stiffness of the four springs should be chosen with the same constant:

(3) k=1.206 N/mm

### 3.2. Arch Mechanism

As mentioned above, the arch function includes two stages: (1) no-load stage, where no contact occurs with any object and motor 2 is idle and drives the thumb adduction to form an arch with the palm, which is ready for sphere grasp, and (2) load stage, where the thumb remains adducted and starts contact with an object and the grasp output increases, whereas the metacarpals extend passively to the initial position. In the sequel, we detail the arch mechanism from the initial position.

#### 3.2.1. No-Load Stage

**Figure 12:**
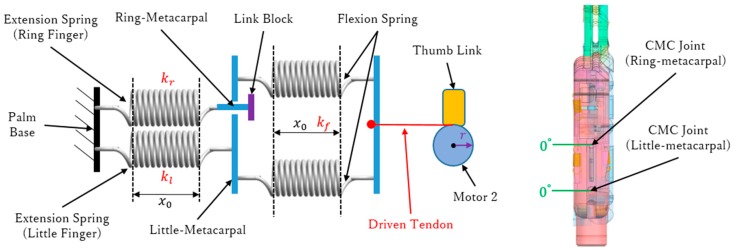
Initial position of the prosthetic hand: (**a**) Diagram and geometry; and (**b**) 3-D model of the hand.

The no-load stage is divided into three steps. Step 1 corresponds to the initial position. The thumb is on the side of the fingers, and motor 2 has not engaged the driven tendon. Hence, the extension and flexion springs are balanced, and the metacarpals do not rotate. Figure 12a shows the diagram and geometry of the symmetrical spring structure, where $k_{l}$ is the stiffness of the extension spring connected to the little-metacarpal, $k_{r}$ is the stiffness of the extension spring connected to the ring-metacarpal, and r is the radius of the motor rotation axis. Figure 12b shows the 3-D hand model in the initial position, where the rotation on every CMC joint is zero. As k is given in Equation (3), we obtain the following:

(4) $$k_{l}=k_{r}=k=1.206\;N/\mathrm{mm}$$

Given that the two flexion springs work together, they can be considered as a parallel spring element, which can be expressed with combined stiffness $k_{f}$:

(5) $$k_{f}=2k=2.412\;N/\mathrm{mm}$$

Initially, motor 2 does not rotate to draw the driven tendon and the symmetrical springs are not triggered. Hence, every spring is in the initial pre-tightening state with same initial stretching $x_{0}$, and the tension of the driven tendon, *T*, is zero.

**Figure 13:**
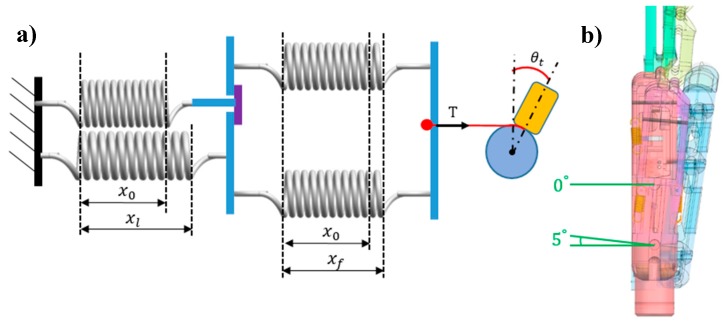
Coupling position of the prosthetic hand: (**a**) Diagram and geometry and (**b**) 3-D model of the hand.

Step 2 corresponds to the coupling position. Motor 2 starts rotating clockwise, and tension *T* through the driven tendon acts on the two flexion springs and is transferred to the extension spring in the little-metacarpal. Thus, the little-metacarpal starts to flex with the rotation of motor 2, and when flexed to 5°, the extension spring in the ring-metacarpal is coupled to that in the little-metacarpal through the link block. Figure 13 illustrates the coupling position, where the little-metacarpal extension spring is connected with the flexion springs in series (Figure 13a). The series spring stiffness can be calculated as follows:

(6) $$\left(k_{l}+k_{f}\right)/k_{l}k_{f}=1.244\;N/\mathrm{mm}$$

where the two flexion springs stretch to $x_{f}$ and the little-metacarpal extension spring stretches to $x_{l}$ at the coupling position. Although the ring-metacarpal extension spring maintains its initial stretching $x_{0}$, it is ready to flex together with the little-metacarpal such that the flexion on the ring-metacarpal is zero, as shown in Figure 13b. Moreover, through geometric parameters d (Figure 10b) and r (Figure 12a), thumb angle $\theta_{t}$ and tension *T* can be calculated, obtaining the following:

(7) $$\theta_{t}=10.175{}^{\circ}$$

(8) T=0.88 N

**Figure 14:**
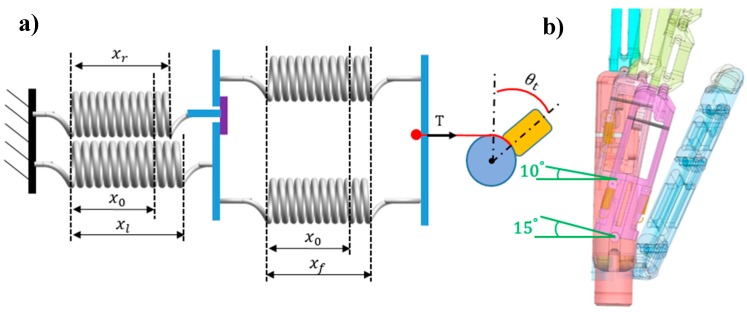
Final arch position of the prosthetic hand: (**a**) Diagram and geometry and (**b**) 3-D model of the hand.

Step 3 corresponds to the final arch position. With motor 2 still rotating, the little- and ring-metacarpals flex together to reach their maximal flexions and to completely form the arch with the thumb. Figure 14a shows that every spring is stretched by the driven tendon. Hence, the four springs work as a unit and the combined stiffness in step 3 can be calculated as follows:

(9) $$\left(k_{l}+k_{r}+k_{f}\right)/\left[k_{f}\left(k_{l}+k_{r}\right)\right]=0.829\;N/\mathrm{mm}$$

Tension *T* is as follows:

(10) T=1.75 N

In Figure 14a, $x_{r}$, $x_{l}$, and $x_{f}$ represent the stretching of the extension springs in the ring- and little-metacarpals and that of the flexion springs, respectively. Figure 14b shows the CMC joint at the final arch state, where the little- and ring-metacarpals flex to their limits of 15 and 10°, respectively, and the thumb angle is as follows:

(11) $$\theta_{t}=30.09{}^{\circ}$$

#### 3.2.2. Load Stage

**Figure 15:**
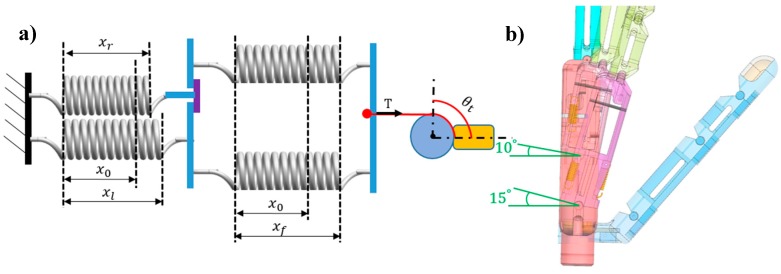
Initial contact position of the prosthetic hand: (**a**) Diagram and geometry and (**b**) 3-D model of the hand.

The load stage is divided into two steps. Step 1 corresponds to the initial contact position. We assume that contact with an object occurs when the thumb rotates to the opposite position with respect to the palm. As shown in Figure 15, thumb angle $\theta_{t}$ is 90°, reaching the maximum adduction at this position. Stretching lengths $x_{r}$ and $x_{l}$ are the same as in the final arch position because both the little- and ring-metacarpals reach their maximal flexion. However, the stretching of the flexion springs continues to increase such that $x_{f}$ becomes larger and tension *T* is as follows:

(12) T=15.15 N

**Figure 16:**
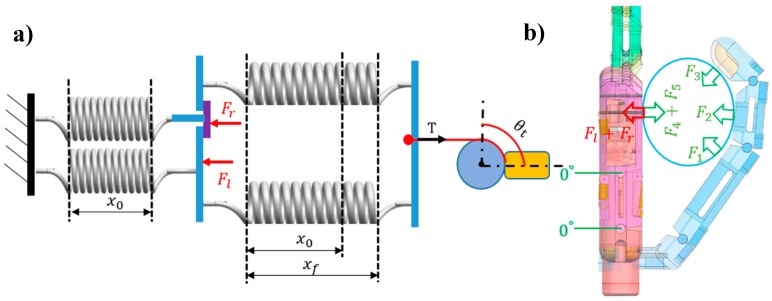
Final extension position of the prosthetic hand when holding an object: (**a**) Diagram and geometry and (**b**) 3-D model of the hand.

Step 2 corresponds to the final extension position. Assume that, after the thumb adducts to 90° under action of motor 2, the metacarpals and the thumb contact with a cylindrical object to form a medium-wrap grasp. Then, motor 3 drives the other three joints (i.e., CMC, MCP, and IP joints) of the thumb to flex them together, resulting in an adaptive grasp to wrap the finger segments around the object. Meanwhile, the little- and ring-metacarpals are pushed back until they return to the complete extension due to the contact force on each of them. This mechanism is shown in Figure 16, where $F_{l}$ is the contact force action on the little-metacarpal and $F_{r}$ is the contact force acting on the ring-metacarpal. In addition, the flexion springs stretch again and stretching $x_{f}$ reaches its maximum. The two extension springs return to the initial state, and thus, both stretch values are $x_{0}$. In this step, tension *T* is as follows:

(13) T=20.24 N

### 3.3. Kinematic and Static Analyses of Arch Function

**Figure 17:**
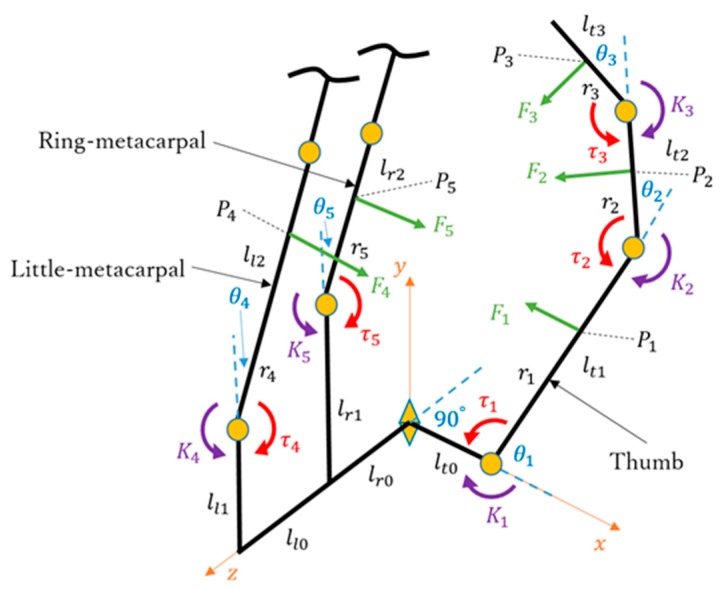
Kinematics and statics model of the prosthetic hand.

Here, we discuss the kinetics and statics changes from the initial contact position (Figure 15) to the final extension position (Figure 16). The kinematics model is shown in Figure 17, where $P_{i}$ and $F_{i}$ (*i* = 1, 2, 3, 4, 5) are the supposed contact points and contact force, respectively, on each phalange and metacarpal. Consider the position vector:

(14) Piti=[ri00]T

The transformation matrix of the contact points on the thumb can be simplified as follows:

(15) Ttii−1=[Cθi−Sθi0lti−1SθiCθi0000100001]

where $C_{\theta i}$ and $S_{\theta i}$ denote the cosine and sine of angle $\theta_{i}$, respectively, and the position vector of each contact point on the thumb can be formulated as follows:

(16) Pi0=Tt10⋯Ttiti−1Piti

where *i* = 1, 2, 3 and $l_{ti}$ is the length of the *ti*th phalange of the thumb.

Next, for the contact points on the ring- and little-metacarpals, the position vector and transformation matrix are given by the following:

(17) P40=Tl20P4l2=[Cθ4Sθ400−Sθ4Cθ40ll1001lr0+ll00001][0r401]

(18) P50=Tr20P5r2=[Cθ5Sθ500−Sθ5Cθ50lr1001lr00001][0r501]

The Jacobian matrix Ji0 can be obtained based on the above equations. Then, the relationship between the contact force and torque vector τ can be expressed as follows:

(19) τ=∑i=15Ji0TFi0

(20) τ=J10TF10+J20TF20+J30TF30+J40TF40+J50TF50

(21) $$\tau =\left[\begin{array}{c} F_{1}r_{1}\\ 0\\ 0\\ 0\\ 0 \end{array}\right]+\left[\begin{array}{c} F_{2}\left(r_{2}+l_{t1}S_{\theta 12}S_{\theta 1}+l_{t1}C_{\theta 12}C_{\theta 1}\right)\\ F_{2}r_{2}\\ 0\\ 0\\ 0 \end{array}\right]+\;\left[\begin{array}{c} F_{3}\left(r_{3}+l_{t2}S_{\theta 123}S_{\theta 12}+l_{t1}S_{\theta 123}S_{\theta 1}+l_{t2}C_{\theta 123}C_{\theta 12}+l_{t1}C_{\theta 123}C_{\theta 1}\right)\\ F_{3}\left(r_{3}+l_{t2}S_{\theta 123}S_{\theta 12}+l_{t2}C_{\theta 123}C_{\theta 12}\right)\\ F_{3}r_{3}\\ 0\\ 0 \end{array}\right]+\left[\begin{array}{c} 0\\ 0\\ 0\\ F_{4}r_{4}\\ 0 \end{array}\right]+\left[\begin{array}{c} 0\\ 0\\ 0\\ 0\\ F_{5}r_{5} \end{array}\right]$$

(22) $$\tau =\left[\begin{array}{ccccc} r_{1} & r_{2}+l_{t1}C_{\theta 2} & r_{3}+l_{t2}C_{\theta 3}+l_{t1}C_{\theta 23} & 0 & 0\\ 0 & r_{2} & r_{3}+l_{t2}C_{\theta 3} & 0 & 0\\ 0 & 0 & r_{3} & 0 & 0\\ 0 & 0 & 0 & r_{4} & 0\\ 0 & 0 & 0 & 0 & r_{5} \end{array}\right]\left[\begin{array}{c} F_{1}\\ F_{2}\\ F_{3}\\ F_{4}\\ F_{5} \end{array}\right]$$

Then, set the matrix on the left of matrix F as matrix Q; therefore, it can be expressed as follows:

(23) τ=QF

(24) $$Q^{-1}\tau =F$$

**Figure 18:**
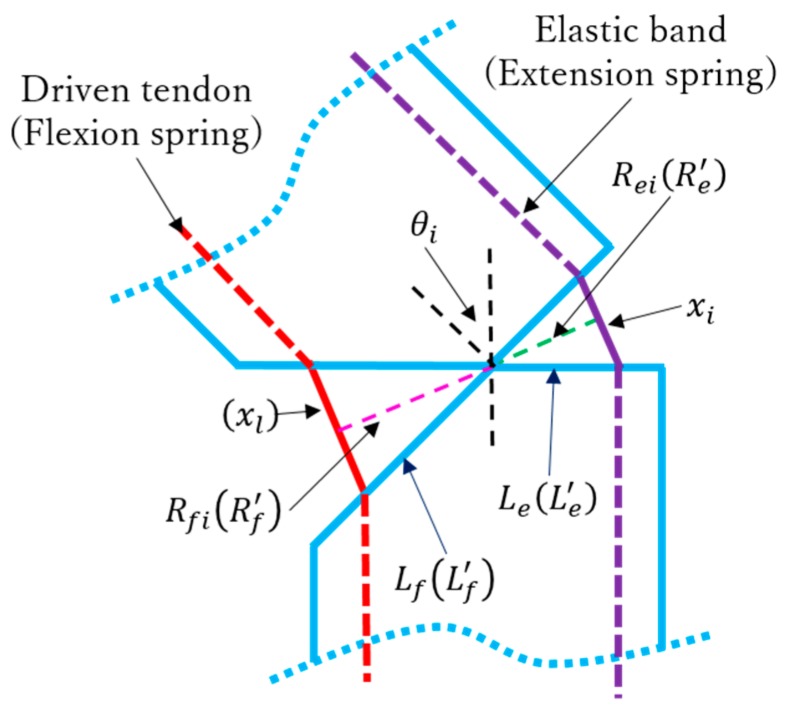
Diagram of joint specifying geometric parameters.

τ is given by the following:

(25) $$\tau =\left[\begin{array}{c} \tau_{1}\\ \tau_{2}\\ \tau_{3}\\ \tau_{4}\\ \tau_{5} \end{array}\right]=\left[\begin{array}{c} \frac{MR_{f1}}{R_{o}}-K_{0}-K_{1}\\ \frac{MR_{f2}}{R_{o}}-K_{0}-K_{2}\\ \frac{MR_{f3}}{R_{o}}-K_{0}-K_{3}\\ K_{l}-K_{4}\\ \frac{27}{52}\left(K_{l}-K_{4}\right)-K_{5} \end{array}\right]$$

with $R_{o}$ being the rotation radius of motor 3, $R_{o}$ = 5 mm, and $K_{l}$ as the spring torque. When *i* = 1, 2, 3, $R_{fi}=L_{f}\cos\left(\pi /4-\theta_{i}/2\right)$ and $K_{i}=kx_{i}R_{ei}=kL_{e}^{2}\sin\theta_{i}$, where $L_{f}=L_{e}$ = 5/20.5. When *i* = 4, 5, $K_{i}=k^{\prime }x_{i}R_{ei}^{\prime }=k^{\prime }L_{e}^{\prime }{}^{2}\sin\theta_{i}$. The geometric parameters are depicted in Figure 18.

Finally, the contact force is given by the following:

(26) Fi0=[FixFiyFiz]T

Thus, we obtain the following:

(27) $$\{\begin{array}{c} F_{1}=\frac{l_{t1}}{r_{1}r_{2}r_{3}}\left[r_{2}\cos\left(\theta_{2}+\theta_{3}\right)-r_{3}\cos\theta_{2}-l_{t2}\cos\theta_{2}\cos\theta_{3}\right]\;\;\;\;\;\;\;\;\;\;\;\;\\ \left[kL_{e}\left(x_{0}+L_{e}\sin\theta_{3}\right)-\frac{ML_{f}}{R_{o}}\cos\left(\frac{\theta_{3}}{2}-\frac{\pi }{4}\right)\right]\\ +\frac{1}{r_{1}r_{2}}\left(r_{2}+l_{t1}\cos\theta_{2}\right)[kL_{e}\left(x_{0}+L_{e}\sin\theta_{2}\right)-\\ \frac{ML_{f}}{R_{o}}\cos\left(\frac{\theta_{2}}{2}-\frac{\pi }{4}\right)]-\frac{1}{r_{1}}\left[kL_{e}\left(x_{0}+L_{e}\sin\theta_{1}\right)-\frac{ML_{f}}{R_{o}}\cos\left(\frac{\theta_{1}}{2}-\frac{\pi }{4}\right)\right]\\ F_{2}=\frac{1}{r_{2}r_{3}}\left(r_{3}+l_{t2}\cos\theta_{3}\right)\left[kL_{e}\left(x_{0}+L_{e}\sin\theta_{3}\right)-\frac{ML_{f}}{R_{o}}\cos\left(\frac{\theta_{3}}{2}-\frac{\pi }{4}\right)\right]-\;\\ \frac{1}{r_{2}}\left[kL_{e}\left(x_{0}+L_{e}\sin\theta_{2}\right)-\frac{ML_{f}}{R_{o}}\cos\left(\frac{\theta_{2}}{2}-\frac{\pi }{4}\right)\right]\\ F_{3}=\frac{1}{r_{3}}\left[\frac{ML_{f}}{R_{o}}\cos\left(\frac{\theta_{3}}{2}-\frac{\pi }{4}\right)-kL_{e}\left(x_{0}+L_{e}\sin\theta_{3}\right)\right]\;\\ F_{4}=\frac{k^{\prime }}{r_{4}\cos2\theta_{4}}\left\{L_{e}^{\text{'}}{}^{2}\sin\theta_{4}+\frac{2}{25}L_{f}^{\text{'}}\left[25L_{f}^{\text{'}}\sin\left(\theta_{4}-\frac{\pi }{12}\right)-157\right]\right\}\\ F_{5}=\frac{k^{\prime }}{26r_{5}}\left\{27L_{f}^{\text{'}}\left[6.28-L_{f}^{\text{'}}\sin\left(\theta_{4}-\frac{\pi }{12}\right)\right]-L_{e}^{\text{'}}\left(13.5\sin\theta_{4}+26\sin\theta_{5}\right)\right\} \end{array}$$

**Figure 19:**
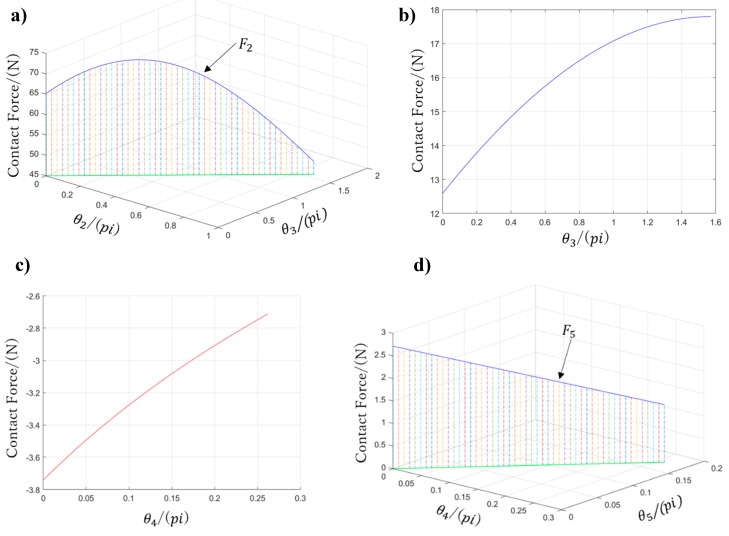
Contact forces on the prosthetic hand: (**a**) *F*_2_ on the proximal phalange of the thumb, (**b**) *F*_3_ on the distal phalange of the thumb, (**c**) *F*_4_ on the little-metacarpal, and (**d**) *F*_5_ on the ring-metacarpal.

Figure 19 shows contact forces F2 to F5, whereas F1 is not plotted given its complex contact condition, which requires all variables $\theta_{1}$, $\theta_{2}$, and $\theta_{3}$.

## 4. Experiments and Results

### 4.1. Finger Motion Verification

**Figure 20:**
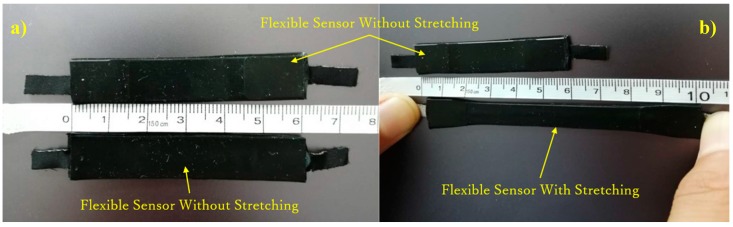
Flexible sensor (**a**) without stretching and (**b**) with stretching.

The proposed prosthetic hand design considers the tendon-driven transmission for the thumb and four fingers. The thumb uses one tendon to perform adaptive grasps, and the four fingers adopt two kinds of tendon mechanisms, enabling both adaptive grasps and a determined grasp trajectory. Given their similarity, we only verified the motion of one of the four fingers to determine the digit ability to perform movements as designed. Flexible sensors (ZM-C-CI-18-01, Z-Mirror, Shenzhen, China) were used in this experiment to measure the joint angles given their high malleability. Figure 20 shows the sensor before and after stretching.

**Figure 21:**
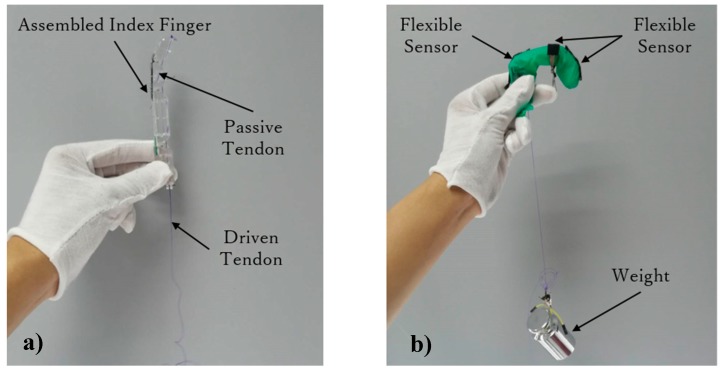
Experimental setup for finger motion verification: (**a**) Assembled index finger and (**b**) experiment with varying weight.

Figure 21 shows the experimental setup for this verification. Figure 21a shows an assembled index finger. Figure 21b shows an image of the experiment, where each finger joint fixes one flexible sensor and a weight draws the tendon to rotate the finger joints. We measured the sensor voltage changes on each finger joint under the action of different weights.

**Figure 22:**
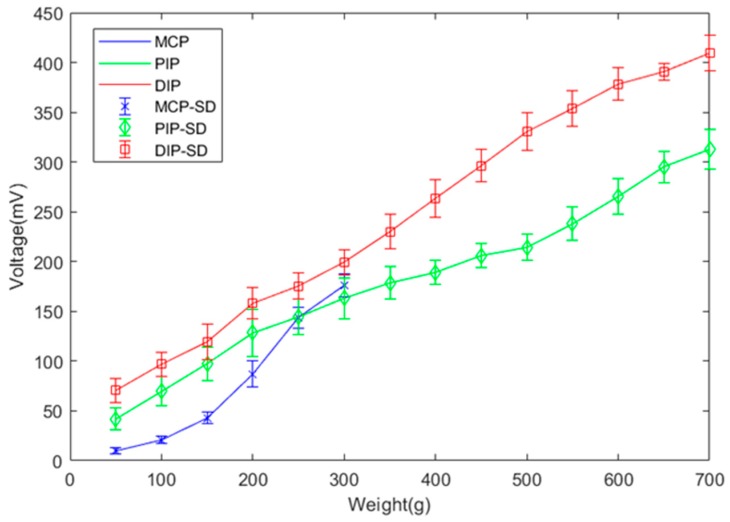
Sensor measurements to verify finger motion at different weights.

For this verification, we used 14 weights starting from 50 g with increments of 50 g, tested ten times. The results of the sensor measurements are shown in Figure 22, expressed with their average value and standard deviation. For weights from 50–300 g, the sensor voltages on the PIP and DIP joints are very similar, as expected by design. Although throughout finger flexion the angle of the PIP and DIP joints should remain the same in theory, inevitable measurement errors are reflected in the results. Consequently, from 350–700 g, the measured data from the PIP and DIP joints diverge.

### 4.2. Arch Motion Verification

**Figure 23:**
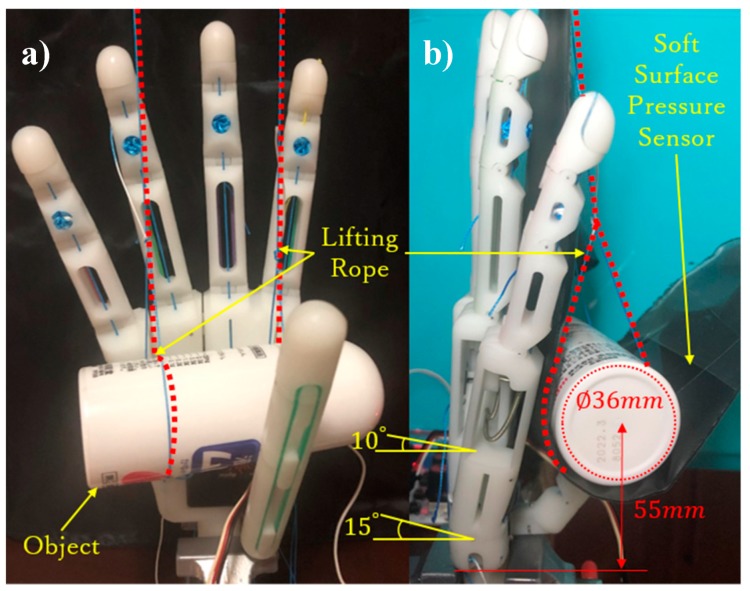
Experimental setup for medium wrap grasp: (**a**) Front view and (**b**) side view during the grasp of a cylindrical object.

To verify the grasping ability for cylindrical objects of the arch analyzed in Section 3, we conducted an experiment performing arch motion. Figure 23 shows a cylindrical object (diameter 36 mm) lifted to a height of 55 mm to minimally allow the hand to contact the object performing the arch posture. Before the experiment, we set the hand to the initial contact position, as shown in Figure 23b, such that the thumb is adducted to 90° and the flexion angles of the little- and ring-metacarpals reach their flexion limits of 15 and 10°, respectively. Then, a 2-mm thick soft-surface pressure sensor (Tactilus bt5010-5101-16×5; Sensor Products Inc., Madison, NJ, USA) was wrapped around the object to measure the pressure acting on each phalange and metacarpal and to obtain the approximate position of the force contact points. It is worth mentioning that the implemented prosthetic hand weighs only 146 g, being notably below the suggested limit of 370 g [34].

**Figure 24:**
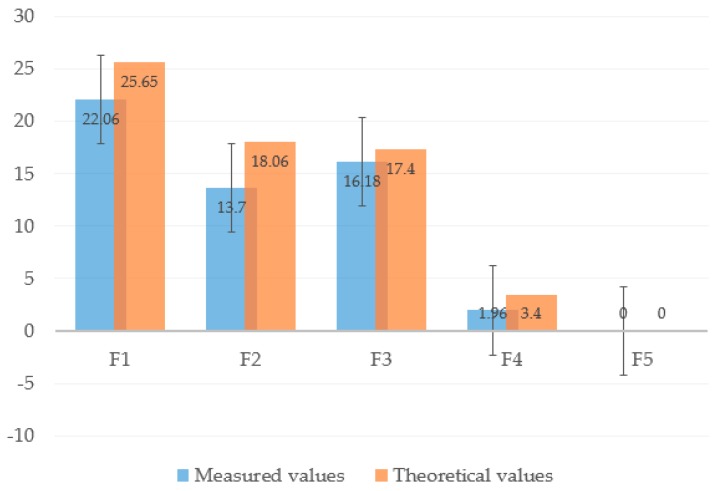
Comparison of forces between measurement results and theoretical values for medium-wrap grasp.

This experiment proceeded as follows. First, as motor 3 enwinds the driven tendon, the thumb begins to flex until the proximal phalange contacts the object, thus stopping the phalange. Then, the IP joint continues flexing until the distal phalange contacts the object. While motor 3 keeps rotating, the CMC joint starts to flex and the contact force transfers through the object to the little- and ring-metacarpals to make them finally extend and release the arch posture. During grasping, we recorded the angle using a digital angle ruler and measured the force on each phalange and metacarpal using the pressure sensor. As measurement or calibration errors are unavoidable, we repeated the experiment five times. According to Equation (27), the theoretical contact force can be calculated by assuming the same grasping condition as for the experiment. Figure 24 shows the comparison between the measurement results and theoretical values, in which the measurements are expressed with their average standard error. Although the measured values are lower than the theoretical ones, they are similar, with the divergence being explained by several influences such as friction loss and measuring errors.

### 4.3. Intuitive EMG-Based Control

**Figure 25:**
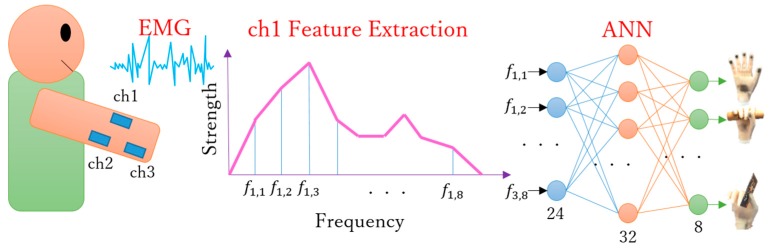
EMG-based control diagram (ANN, artificial neural network).

After completing the abovementioned motion verifications, we focused on the implementation of an intuitive control for the prosthetic hand [34]. For this experiment, we tested the operability and motion performance using real-time EMG-based control, as illustrated in Figure 25. First, EMG signals were collected from a subject’s forearm and then sampled to 2 kHz via analog-to-digital conversion using a microcomputer (SH72544R, Renesas Electronics, Tokyo, Japan). Next, eight-dimensional feature vectors were obtained through the fast Fourier transform in one independent channel. As there are three independent channels, 24-dimensional feature vectors were obtained and used as training or validation data for an artificial neural network. After training, the network returned one kind of hand motion through pattern recognition from real-time signals. Finally, the prosthetic hand performed the motion according to the identified motion [35,36,37,38].

**Figure 26:**
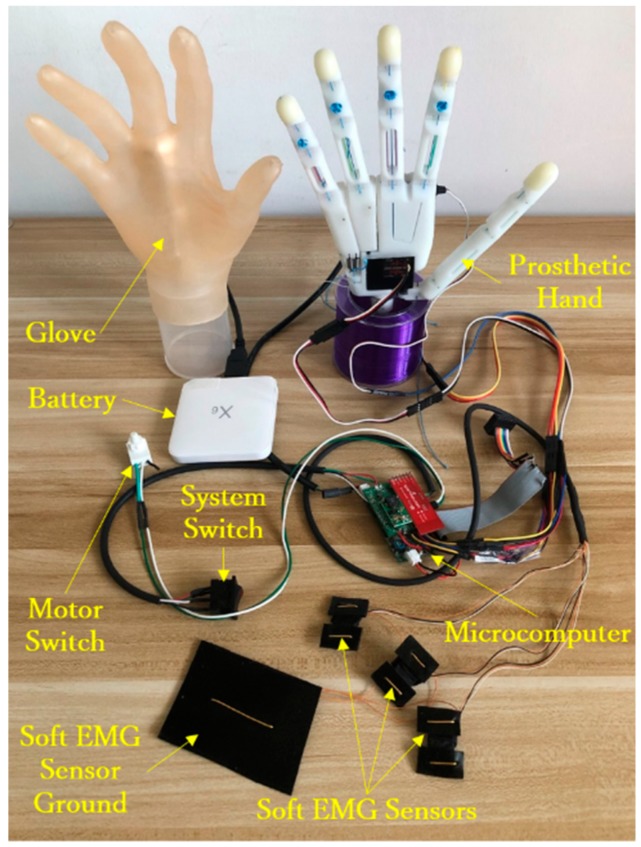
Experimental setup to test different types of grasps.

**Figure 27:**
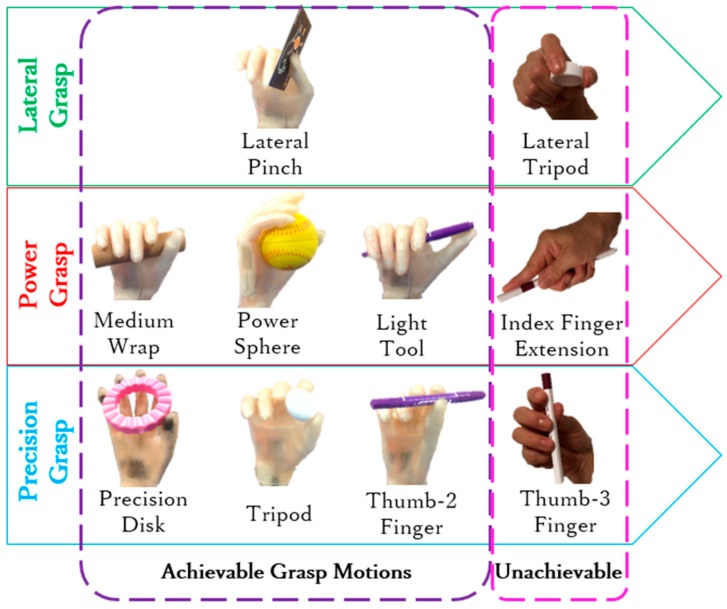
Ten most frequent types of grasps for experimental testing (adapted from Reference [13]).

Figure 26 shows the devices employed for this experiment. A healthy adult male (age: 35 years, right-handed) participated in this study. EMG sensors were attached to his right forearm. The subject was asked to perform grasping tasks with objects of different shapes and sizes, based on the ten frequent grasps shown in Figure 1. Figure 27 illustrates the experiment results, in which the ten most frequent grasps are classified as either achievable or unachievable. All the achievable grasp motions are shown with the real photos taken during the experiment, and the unachievable grasp motions are shown with images. For the achievable grasp motions, lateral pinch, light tool, tripod, and thumb-2 finger can be regarded as regular motions because they are the most achievable motions even without the arch function, and can thus be performed with existing prosthetic hands. Unlike existing prostheses the arch function allows the proposed hand to achieve the power sphere and precision disk naturally, mimicking the human hand surrounding objects. Furthermore, as expected from design, the arch posture can be passively released, switching between sphere and medium wrap grasps like the human hand, and thus the medium wrap is also easily achievable. The remaining kinds of grasp motions, lateral tripod, index finger extension, and thumb-3 finger, are unachievable as each of the four fingers cannot provide adaptive flexion respectively. Still, the proposed prosthesis allows performing multiple types of hand grasp motions, accounting for 70% of the ten most frequent grasps. Remarkably, given the arch function, power sphere and precision disk can be fulfilled, outperforming current prosthetic hands. Moreover, the arch posture can be released passively, and hence the medium wrap is also available.

**Figure 28:**
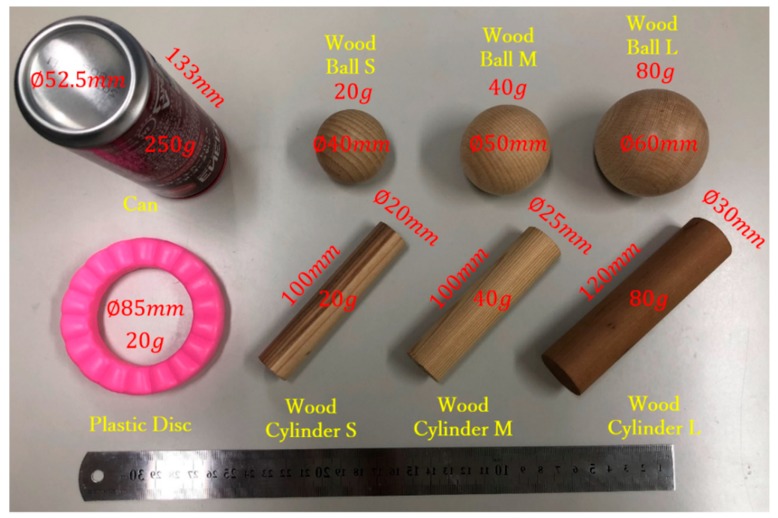
Objects used in the Pick-and-Place experiment.

**Figure 29:**
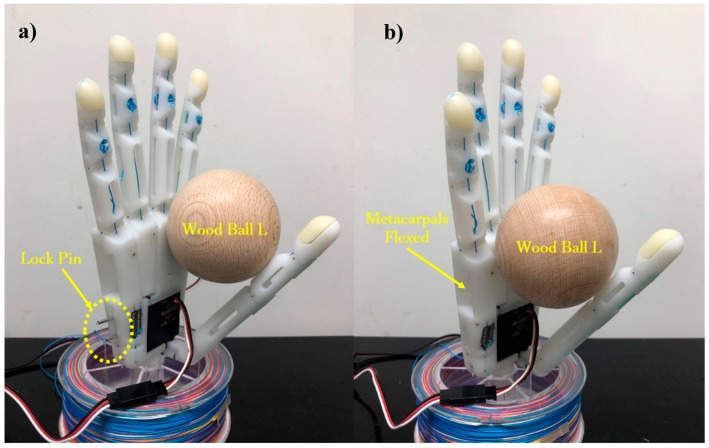
Modes of prosthetic hand during Pick-and-Place experiment: (**a**) without the arch function, the ball is held by the thumb and palm; (**b**) with the arch function, the metacarpals flex to form an arch with the thumb.

**Figure 30:**
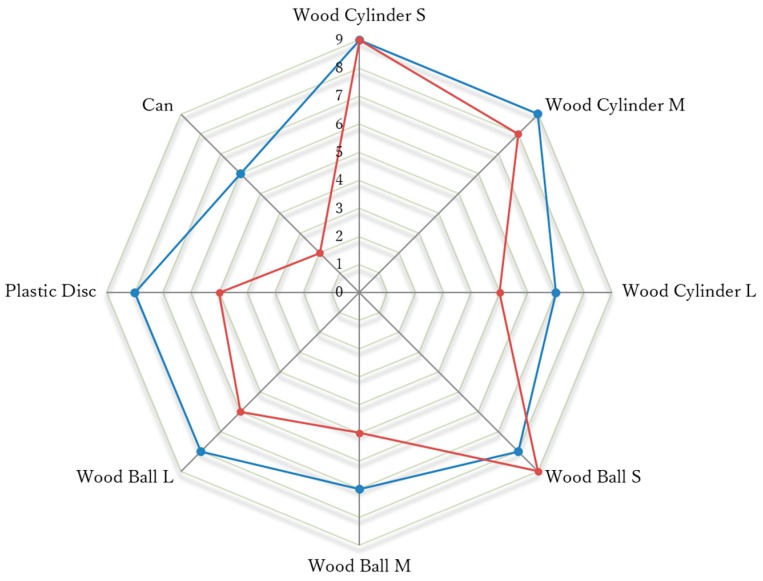
Pick-and-Place experiment result.

Then, we conducted a Pick-and-Place experiment to verify the usefulness of the arch function during grasps. Figure 28 shows the objects used in this experiment along with their weights, sizes, and shapes. For the experiment, we marked two 15 × 15 mm^2^ squares on a table separated by 300 mm. Then, for each object, the prosthetic hand was controlled to pick up the object from one marked area and move it to the other. The subject was required to perform the task repeatedly for 30 s. If the object did not fall from the prosthesis during the translation, it was considered a successful task. Figure 29 shows two modes of the prosthetic hand: (a) little-and ring-metacarpal locked by two pins, such that the hand cannot form an arch for grasping; and (b) using the arch function, the metacarpals flex to form an arch with the thumb during grasp. To compare the task completion with and without the arch function, the subject performed the Pick-and-Place experiment under each mode, and repeated the tasks five times. Figure 30 show the results from this experiment as the average among the five trials. The grasping performance notably improves with the arch function, especially for heavy objects such as the can, possibly by the increased contact area obtained from the arch function, which improves the grasping stability.

**Figure 31:**
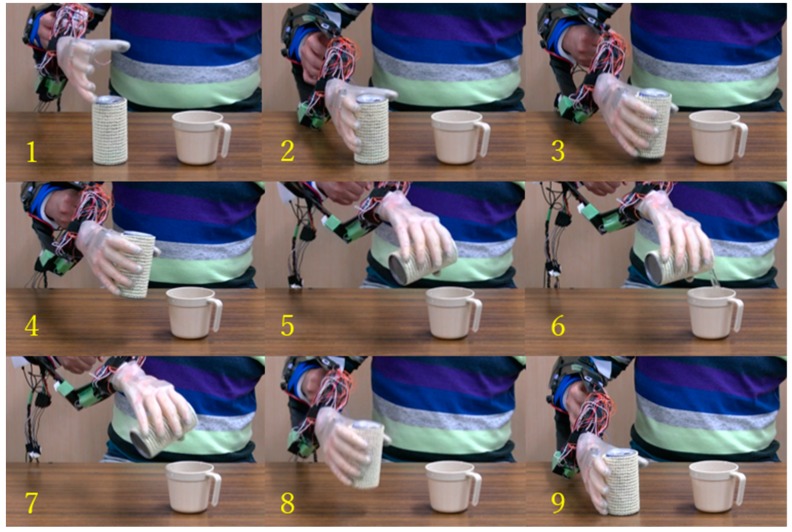
Grasping to perform an activity of daily living.

Finally, we verified the grasp performance of the prosthetic hand covered with a silicon glove by performing an activity of daily living. The prosthetic hand was equipped on the right forearm of a subject via a socket. The subject was asked to perform several grasp tasks to pick up some objects. Figure 31 shows the sequence of one task, where the whole process for the subject to pick up a water can from a table, pour water on a cup, and return the can to a table is depicted. From a qualitative evaluation, we determined that the experiment was successfully performed, thus verifying the practical capabilities of the proposed prosthetic hand.

## 5. Conclusions

We propose an adaptive multi-finger prosthetic hand that has human-like appearance and size. The four fingers move as a unit to provide both adaptive grasping and a determined trajectory for grasping, whereas the thumb is totally independent and contributes to the adaptive grasp (flexion) and active rotation (adduction/abduction). An important design innovation is the palm with movable metacarpals instead of being a rigid block like in conventional prostheses. Hence, the palm is capable of forming an arch with the thumb, resembling human hand motions much more naturally. In addition, the arch posture can be released passively, like in the human hand, and allows switching between sphere and medium wrap grasps according to the situation.

The proposed prosthetic hand design aims not only at releasing hand joints for improved flexibility but also at complying with weight and size requirements for prostheses. The prosthetic hand consists of only three small actuators embedded in the palm. Although the prosthetic hand has a comparable size to the adult human hand, it only weighs 146 g. We analyzed the motion mechanism through the kinematics and statics model and demonstrated the grasping performance through motion verifications and a control experiment including activities of daily living. Overall, the developed prosthetic hand can provide a variety of hand grasps, accounting for 70% of the 10 most frequent grasps and covering all the main grasp categories, namely, power, lateral, and precision grasps.

As future work, we will focus on optimizing the hand structure design to achieve more types of grasps and higher mobility. Moreover, we will aim to improve the actuation system to obtain a larger output force, to deal with losses on the tendon-driven transmission, and to endow the prosthesis with sensors for better control.
